# Multiplicity of Carbapenemase-Producers Three Years after a KPC-3-Producing *K. pneumoniae* ST147-K64 Hospital Outbreak

**DOI:** 10.3390/antibiotics9110806

**Published:** 2020-11-13

**Authors:** Ana Margarida Guerra, Agostinho Lira, Angelina Lameirão, Aurélia Selaru, Gabriela Abreu, Paulo Lopes, Margarida Mota, Ângela Novais, Luísa Peixe

**Affiliations:** 1 UCIBIO, Faculdade de Farmácia, Universidade do Porto, 4050-313 Porto, Portugal; anamg.guerra@gmail.com; 2 Centro Hospitalar Vila Nova de Gaia/Espinho, 4434-502 Vila Nova de Gaia, Portugal; lira@chvng.min-saude.pt (A.L.); angelinalameirao@chvng.min-saude.pt (A.L.); aurelia.selaru@chvng.min-saude.pt (A.S.); gabriela.abreu@chvng.min-saude.pt (G.A.); microlopes@chvng.min-saude.pt (P.L.); mmota@chvng.min-saude.pt (M.M.)

**Keywords:** carbapenemase-producing Enterobacterales, KPC, carbapenem, multidrug resistance, nosocomial

## Abstract

Carbapenem resistance rates increased exponentially between 2014 and 2017 in Portugal (~80%), especially in *Klebsiella pneumoniae*. We characterized the population of carbapanemase-producing Enterobacterales (CPE) infecting or colonizing hospitalized patients (2017–2018) in a central hospital from northern Portugal, where KPC-3-producing *K. pneumoniae* capsular type K64 has caused an initial outbreak. We gathered phenotypic (susceptibility data), molecular (population structure, carbapenemase, capsular type) and biochemical (FT-IR) data, together with patients’ clinical and epidemiological information. A high diversity of Enterobacterales species, clones (including *E. coli* ST131) and carbapenemases (mainly KPC-3 but also OXA-48 and VIM) was identified three years after the onset of carbapenemases spread in the hospital studied. ST147-K64 *K. pneumoniae*, the initial outbreak clone, is still predominant though other high-risk clones have emerged (e.g., ST307, ST392, ST22), some of them with pandrug resistance profiles. Rectal carriage, previous hospitalization or antibiotherapy were presumptively identified as risk factors for subsequent infection. In addition, our previously described Fourier Transform infrared (FT-IR) spectroscopy method typed 94% of *K. pneumoniae* isolates with high accuracy (98%), and allowed to identify previously circulating clones. This work highlights an increasing diversity of CPE infecting or colonizing patients in Portugal, despite the infection control measures applied, and the need to improve the accuracy and speed of bacterial strain typing, a goal that can be met by simple and cost-effective FT-IR based typing.

## 1. Introduction

The dissemination of carbapenemase-producing *Enterobacterales* (CPE) has been causing serious concerns in many EU countries due to the limited therapeutic options and ineffective infection control policies [1,2]. Portugal is one of the EU countries where the rates of carbapenems resistance amongst invasive isolates has been alarmingly rising (~80% between 2014 and 2017), especially among *Klebsiella pneumoniae* for which reported rates in 2018 were 11.7% [3,4]. Reporting of CPE from national laboratories is mandatory since 2013 and recommendations to control CPE spread that include identification of rectal carriers and improvement of infection control policies in the hospital setting have been officially launched in 2017 [5]. 

Available data on molecular epidemiology and population structure of CPE populations in the country represent scattered snapshots on particular species and/or small institutions. The ST147 *K. pneumoniae* clone exhibiting K-type K64 has been one of the first responsible for KPC-3 spread in diverse healthcare settings in the north of Portugal [6,7]. It was responsible for the first large outbreak of CPE in a reference hospital from the north of Portugal in 2015 [8] and it has been described in other regions [9,10]. Analysis of recent CPE collections is still scarce and there is limited analysis on risk factors and the effect of infection control policies in a single institution. 

Considering that precise and timely bacterial typing information can make the difference in the effectiveness of infection control measures, we have recently proposed a quick and cost-effective approach for accurate *K. pneumoniae* typing based on an in-house database of Fourier-Transform Infrared (FT-IR) discriminatory spectra [11]. 

In the present study, we aim to understand the evolution of CPE population in a central hospital from northern Portugal three years after the onset of carbapenemases spread [8] in order to identify critical factors for the increasing burden of CPE in hospitalized patients. Moreover, we use this collection to evaluate the performance of our method for accurate subtyping of *K. pneumoniae* contemporaneous isolates. 

## 2. Results

### 2.1. The Incresasing Diversity of CPE Population

Carbapenemase production was confirmed in 120 out of the 128 (94%) recovered isolates, that were subsequently characterized. The remaining ones were possible ESBL producers with permeability defects. These 120 isolates were identified in rectal swabs (*n* = 86) and clinical samples (*n* = 34) from 114 patients. Multiple *Enterobacterales* species were identified in either clinical samples or rectal swabs, though *K. pneumoniae* was by far the most frequent (*n* = 98; *n* = 66 from rectal swabs and *n* = 32 from clinical samples) (Table 1). While nearly all isolates from infection belonged to *K. pneumoniae* (n = 32/34; 94%), the isolates from fecal samples presented a greater diversity, including mostly *K. pneumoniae* (*n* = 66/86; 76.7%), but also *E. coli* (*n* = 10/86; 11.6%), different *E. cloacae* complex species including *E. asburiae* and *E. hormaechei* (*n* = 5/86; 5.8%), *K. oxytoca* (*n* = 4/86; 4.7%), and *Citrobacter freundii* complex (*n* = 1/86; 1.2%), the latter two appearing exclusively in colonization samples. *E. coli* was identified mainly from rectal swabs (*n* = 10/11; 91%). Most isolates produced KPC-3 (*n* = 118/120; 98.3%) including all *K. pneumoniae*, but OXA-48 and VIM-1 were also occasionally detected in 1 *E. coli* clinical isolate and 1 *E. hormaechei* identified as a gastrointestinal colonizer, respectively. *mcr* genes were not detected. 

Simultaneous rectal carriage (in the same sample) by two different species of CPE (*K. pneumoniae* and *E. coli*) or two different *K. pneumoniae* clones was found in four and one patients, respectively. In all cases, both species/clones were KPC-3 producers, which may indicate the interspecies transfer of a KPC-3-encoding plasmid. In the only case where isolates from urine and blood were recovered from the same patient, they had identical phenotype and genotype.

### 2.2. Epidemiological and Clinical Data of Patients Carrying CPE Isolates

Patient’s age ranged from 20 years to 96 years, with mean age of 74 ± 14 years (median 78 years) and female patients were predominant (59% female versus 41% male). They were mostly located in medical (*n* = 55, 48%) or surgical (*n* = 51; 45%) wards, whereas seven (6%) of them were patients attending the emergency room, and 1 (0.9%) was at intensive care unit. Clinical isolates (*n* = 34) were identified mostly at surgical units (*n* = 17/34; 50%), whereas isolates from rectal screenings were obtained mostly from medical wards (*n* = 50/86; 58%). 

The clinical isolates were identified in urine (*n* = 20; 57%) and pus (*n* = 7; 21%) but also in blood (*n* = 3; 9%), biologic liquid (*n* = 2; 6%), or respiratory samples (*n* = 2; 6%). The majority of them were *K. pneumoniae* (*n* = 32/34; 94%), but also *E. cloacae* (*n* = 1/34; 3%) or *E. coli* (*n* = 1/34; 3%). Clinical isolates were identified in 4 medical units (internal medicine was the most frequent, 55%) and 7 surgical units (many from women’s surgery department, 35%). Only ten of the patients with clinical isolates were primarily diagnosed with infectious diseases at ICU, emergency or medical units, whereas in many of the other patients, infection occurred during hospitalization for a median of 30 days (Table 2). Most infected patients (93%) were previous colonized (0–421 days, mean 44 days), most of them in the month previous to infection. Isolates from outpatients (*n* = 6; 74–94 years old) were collected from urine (*n* = 5/7; 71%) and blood (*n* = 2/7; 2.9%). Three of these outpatients were previous CPE carriers from previous hospitalizations, and two of the other three patients had been hospitalized in the last month, all of this suggesting nosocomial acquisition. Most of these patients (70%) received antibiotherapy in the three months prior to infection, even most (87%) of those that were not diagnosed with infectious diseases.

Isolates from rectal swabs were collected from patients at 9 medical (*n* = 56; 65%) or 7 surgical units (*n* = 29, 34%). Internal medicine (*n* = 24; 43%) and unit III (a satellite hospital at a 23Km distance, *n* = 10; 18%) were the most frequent units within the medical department, whereas orthopedics and women’s surgery (*n* = 8; 23% each) were the most common units within surgical departments. Of highlight, a portion of isolates (*n* = 22, 26%) from both medical and surgical units were recovered from 20 patients identified as CPE carriers that were selected for decolonization protocol by gentamicin use. Eight of them were being followed for long periods of time (between 2016 and mid 2017). From these, most (87.5%) had at least one negative CPE screening, on average, 208 days after initial gentamicin treatment though 3 negative rectal screenings are needed to consider a successful decolonization. 

In total, from the 81 patients screened for CPE in rectal swabs, 26 (32%) patients had positive screenings from previous hospitalizations at this hospital. From the 42 (52%) *de novo* CPE carriers, most had long (>14 days) hospitalization periods (*n* = 22; 52%) or had had previous antibiotherapy treatments (*n* = 17; 40%). The remaining 13 screening samples belonged to patients (16%) with no history of screenings. 

In relation to antibiotic exposure, 39 from the 42 *de novo* colonized patients (93%) were exposed to antimicrobials in the previous three months. The main antibiotics administered were piperacillin plus tazobactam (27%), amoxicillin plus clavulanic acid (20%), and broad spectrum cephalosporins (10% ceftriaxone).

*K. pneumoniae* isolates were distributed among all origins (50% medical, 41% surgical, 8% emergency room, 1% ICU), whereas six out of the 11 *E. coli* isolates were recovered from patients under decolonization protocol (*n* = 6/11, 55%) and *E. cloacae* complex, *K. oxytoca*, and *C. freundii* complex were identified mainly or exclusively in surgical units (83%, 75%, and 100%, respectively).

### 2.3. Multiplicity of Clonal Lineages and Emergence of Other International High-Risk Clones

Using our FT-IR spectral workflow and database (11), we were able to predict the capsular type of 94% of the *K. pneumoniae* isolates (6 isolates were not predicted by our model). From those, we grouped isolates in 9 clones. Four of them had identity with capsular types included in our model and identified as K64 (ST147), KL112 (ST15), K19 (ST15), and KL105 (ST11). The remaining five were predicted as not belonging to any of the 19 classes (K-types) included in our model. Indeed, they were subsequently identified as K9 (ST22), KL27 (ST392), KL102 (ST307), KL15/KL17/KL51/KL52 (not typed by MLST), or K53 (not typed by MLST). Only two isolates (2%) were incorrectly predicted (Table 3). 

Three years after the initial outbreak, ST147-K64 is still the predominant *K. pneumoniae* clone (55% overall) responsible for 62.5% of CPE infections and also the most frequent *K. pneumoniae* colonizer (52%). They were recovered from patients at several medical or surgical units (50% vs 39%, respectively) and also in patients at emergency room (11%). The second most represented clone was ST392-KL27 (20.4%) that was mainly identified in medical wards (85%) in February 2018 (Table 3). A diversity of other clones including ST22-K9 (5.1%), ST307-KL102 (4.1%), ST15-K19 (3%), ST15-KL112 (2%), or others were identified as colonizers before or after infections (Table 3).

The carbapenem resistant *E. coli* isolates belonged to distinct phylo-groups, being B2 the most prevalent one (*n* = 5; 46%), followed by groups A (*n* = 2; 18%), E (*n* = 2; 18%), B1 (*n* = 1, 9%), and F (*n* = 1, 9%). One of the B2 *E. coli* producing KPC-3 belonged to the worldwide disseminated clone ST131 (Table 4). 

### 2.4. A Variety of Multidrug Resistance Profiles

All isolates showed multidrug resistance (MDR) phenotypes and were resistant to third generation cephalosporins (100%) and beta-lactam/beta-lactamase inhibitors (100%) and the vast majority was resistant to ciprofloxacin (90%). All carbapenemase producers tested were nonsusceptible to ertapenem (97% resistant, 3% intermediate) and demonstrated susceptible (S), intermediate (I), or resistance (R) phenotypes to imipenem (61% R, 32% I, 7% S) and meropenem (54% R, 31% I, 15% S). Conversely, amikacin (94%), as well as tigecycline (95%), tetracycline (80%), chloramphenicol (80%), or fosfomycin (78%) had the highest susceptibility rates. 

Susceptibility patterns varied according to species and clone. Besides third generation cephalosporins, carbapenems and ciprofloxacin, ST307 and ST22 *K. pneumoniae* were resistant or intermediate to all aminoglycosides (except amikacin), and ST22 additionally to trimethoprim/trimethoprim-sulfamethoxazole and chloramphenicol. ST147 and ST392 were variably resistant to aminoglycosides (ST147-29%; ST392-57%) other than amikacin, trimethoprim-sulfamethoxazole (ST147-46%; ST392-43%). The clinical *E. cloacae* isolate was the most susceptible in this study (susceptibility to all aminoglycosides, ciprofloxacin, chloramphenicol, tetracycline, tigecycline, trimethoprim, and trimethoprim-sulfamethoxazole). On the contrary, the clinical OXA-48-producing *E. coli* was susceptible only to meropenem/imipenem, tetracycline, tigecycline, and chloramphenicol. 

## 3. Discussion

This study highlights a high multiplicity of species, clones, and carbapenemases three years after the initial outbreak by KPC-3-producing *K. pneumoniae* ST147, irrespective of the infection control measures applied. This information, together with epidemiological and patients’ data is critical to understand galloping carbapenems resistance rates in our country and support the revision of infection control measures. To our knowledge, this is the first study providing a comprehensive analysis of the molecular epidemiology of CPE in a single institution in our country in two different time-points of CPE spread, towards a comprehension of factors driving their extraordinary increase. The multiplicity of carbapenemase-producing clones and *Enterobacterales* species, together with data from previous studies in Portugal [7,9,10,12], highlights successful horizontal transfer of carbapenemases that might occur during gastrointestinal colonization, which represents an additional challenge for infection control. 

In contrast to previous studies [9,10,12], ST147-K64 *K. pneumoniae* is still the predominant clone in different healthcare settings in our area. Its identification in patients that are colonized or infected in multiple occasions throughout time, and its dispersion in other community-based healthcare-associated settings suggests possible reintroductions of this highly transmissible clone in the hospital. The emergence of other KPC-3-producing high-risk *K. pneumoniae* clones with recognized worldwide expansion (ST392, ST307) and additional enhanced virulence and/or antibiotic resistance (ST307, ST22) is of concern. The ST392 clone has been recently reported in other countries (China, Italy, Mexico) [13,14,15] but to our knowledge this is the first study to describe KPC-3-producing *K. pneumoniae* ST392 isolates in Portugal. Although detected in small numbers, the identification of 4 ST307 isolates producing KPC-3 among medical and surgical units is of great concern because of its higher resistance profile and the recognized virulence potential (high resistance to complement-mediated killing) of this high-risk clone reported worldwide [16,17]. Our data confirms the absence of ST258 *K. pneumoniae* in our country, a clone that is well-established in other neighboring European countries [18,19]. Furthermore, the detection of ST131 *E. coli* producing KPC-3 especially as rectal colonizers poses a major threat to public health, since it has the potential to cause widespread resistance to carbapenems in the community setting.

We demonstrated an excellent performance of our FT-IR based approach for subtyping multidrug resistant *K. pneumoniae* populations, that was corroborated by reference genotypic molecular methods. Isolates relationships’ were correctly established for 98% of the isolates and, for some of them, it was possible to identify previously circulating *K. pneumoniae* lineages (e.g., ST147-K64, ST15-K19) for which epidemiological data and resistance patterns are well-known [7,11]. This information is useful for infection control teams in order to more quickly and effectively implement measures to control the spread of problematic lineages and eventually guide antibiotic therapy. Thus, the high accuracy of the method, together with its simplicity and extremely short time-to-response (we can provide results from 1-3h after standardized growth conditions) represent ideal features for routine implementation and a real-time support to infection control in the context of outbreak detection [8] or epidemiological surveillance (this study). New K-types and clones were introduced in our spectral database for further improvement of the model and correct predictions of future unknown isolates. 

Besides establishing the potential for diversification of the CPE population, the data obtained in this study were critical to support more targeted infection approaches in the most affected units (medical and surgical wards) and to control the spread of ST392 in medical units (data now shown). The high number of CPE in internal medicine (22.5%) is in agreement with previous data [9], and occurs due to the higher burden of elderly patients, long hospitalization periods and the higher frequency of invasive procedures [20]. Besides, reinforcement of medical equipment, surfaces’ cleaning and contact precautions between patients and healthcare professionals is critical to control the situation [21]. From the data presented, it can be concluded that nearly all *de novo* CPE carriers (n = 43; 96%) became colonized by CPE after hospital admission which strongly supports nosocomial acquisition. Previous colonization and exposure to antibiotics in the previous three months were also recognized as risk factors for development of CPE infections, as previously [20]. Patients maintained carrier status over 200 days, raising questions about the effectiveness of routine decolonization protocols that would need to be evaluated in a longer period and a larger sample. In any case, it is known that available data varies according to decolonization strategy and the benefits are still questionable, and for that reason routine decolonization of CPE is not recommended [22].

In conclusion, this work highlights an increasing diversity of CPE infecting or colonizing hospitalized patients in a central hospital from the north of Portugal, even after implementation of recommended infection control measures guided by clinical and epidemiological data and routine rectal screening results. These results alert for the need to improve the accuracy and speed of bacterial strain typing information, a goal that can be met by simple and cost-effective FT-IR based typing. We strongly believe that, together with epidemiological data and antibiotic resistance patterns, it can be an asset to support infection control and patient’s management in real-time. 

## 4. Materials and Methods 

### 4.1. Study Design and CPE sample

The hospital studied is a central and reference hospital located in the north of Portugal that includes 3 units and 1 rehabilitation center that serves 700.000 inhabitants of that region. It contains capacity for 580-beds, and offers healthcare specialties within medical, surgical, and emergency (adults and pediatric) wards as well as attendance to outpatients. 

In this hospital, the first noticed outbreak by CPE occurred in 2015 by a multidrug resistant KPC-3 producing *K. pneumoniae* ST147 clone with capsular (K)-type 64, that caused the death of 3 patients [8]. From then, the hospital implemented standard infection control measures (including active screening, isolation and contact precautions for infected or colonized patients) that are still in use; however, CPE increased throughout time. 

At the hospital, suspected CPE isolates (*n* = 128) identified from either clinical samples (*n* = 35, November 2017-February 2018) or from rectal swabs (*n* = 93 in February 2018) from hospitalized patients were collected. Screenings of CPE in rectal swabs were performed: (i) at admission in high-risk patients, i.e. those with hospitalizations longer than 72 h in the last 6 months or transferred from another healthcare institution; (ii) at 48 h of hospitalization when the result is negative at admission; (iii) each 7 days of hospitalization if the prior result is negative. 

At admission, CPE colonizers were detected by molecular biology using Xpert CarbaR (Cepheid), whereas subsequent screenings on hospitalized patients were performed using a cultural method. Rectal swabs (positive for Xpert CarbaR and subsequent screenings) were plated on the chromogenic agar chromID Carba Smart (bioMérieux) for recovery of carbapenem resistant isolates. Presumptive *Enterobacterales* isolates (pink colonies for *E. coli* and bluish-green colonies for *Klebsiella*, *Enterobacter*, *Serratia* or *Citrobacter* sp.) were selected for further characterization (>1 isolate per sample was studied when representing different morphotypes or species). 

All patients with positive results were transferred to cohort areas and submitted to infection control measures. We collected patients’ data (age, gender, underlying conditions), hospitalization, and rectal screening history and previous antibiotherapy. 

### 4.2. CPE Identification and Antibiotic Susceptibility Testing 

Isolates from clinical samples were identified and preliminarily tested for antibiotic susceptibility by VITEK^®^2 system (bioMérieux). When necessary, species identification was confirmed by MALDI-TOF MS (VITEK MS, bioMérieux) and further sequencing of *leuS* for speciation within *Enterobacter cloacae* species [23]. 

Extended antibiotic susceptibility profiles were subsequently obtained by disk diffusion for 20 antibiotics (cefotaxime, ceftazidime, cefepime, ertapenem, meropenem, imipenem, amoxicillin-clavulanic acid, piperacillin-tazobactam, amikacin, tobramycin, gentamicin, kanamycin, netilmicin, ciprofloxacin, tetracycline, tigecycline, fosfomycin, trimethoprim, trimethoprim-sulfamethoxazole, and chloramphenicol), according to EUCAST guidelines (www.eucast.org) and CLSI [24].

### 4.3. Molecular Characterization of CPE Producers 

Carbapenemase production was confirmed by the Blue-Carba test [25] and the type of carbapenemase was identified by PCR directed to the most frequent carbapenemase gene families (*bla*_KPC_, *bla*_OXA-48_, *bla*_IMP_; *bla*_VIM_, *bla*_NDM_) and further sequencing [7,26]. *mcr* genes (*mcr-1* to *mcr-5*) were sought by a multiplex PCR previously described [27] in representative isolates from different clones and species. 

### 4.4. FT-IR for Subtyping of K. pneumoniae Carbapenemase Producers

The relationship between *K. pneumoniae* isolates was established by Fourier Transform Infrared (FT-IR) spectroscopy using our previously described workflow [11]. Briefly, bacterial spectra were acquired in standardized conditions and compared with those from our in-house *K. pneumoniae* database (including 19 international clones/K-types) for identification of capsular (K) types and because of established K-type and clone relationships, presumptive clonal identification. These FT-IR-based assignments were confirmed by PCR and sequencing of *wzi* gene [11,28] and multi-locus sequence typing (MLST) using the seven housekeeping genes (*gapA*, *infB*, *pgi*, *mdh*, *phoE*, *rpoB*, *tonB*) proposed in Pasteur MLST scheme in representative isolates of clones with >3 isolates each (https://bigsdb.pasteur.fr/klebsiella/klebsiella.html). 

*E. coli* phylogenetic groups were identified by PCR, according to the quadriplex method described by Clermont et al. [29]. *E. coli* ST131 was presumptively identified by a specific PCR in isolates belonging to the phylogenetic group B2 and *fumC* sequencing [30]. 

## Figures and Tables

**Table 1:** Enterobacterales species identified as carbapenemase producers.

Microorganism	Colonization		Infection		Total	
	No.	%	No.	%	No.	%
***Klebsiella pneumoniae***	66	77	32	94	98	82
***Escherichia coli***	10	11	1	3	11	9
***Enterobacter cloacae*** **complex**	5	6	1	3	6	5
***Klebsiella oxytoca***	4	5	-	-	4	3
***Citrobacter freundii*** **complex**	1	1	-	-		1
**Total**	86	100	34	100	120	100

**Table 2:** Epidemiological data on clinical CPE isolates.

Unit	Patient Nº (Sex/Age)	Clone (MLST)	Species	Isolation Date	Diagnosis of Infectious Diseases	Product	Previous Antibiotherapy (3 months)	Previous Hospitalizations	Duration of Hospitalization	Previous Colonization ^a^
Emergency	2 (M/79)	ST147	*K. pneumoniae*	19/11/17	N	U	Y	Y (this hospital, 2 months)	7 days	+ (2 months)
Emergency	3 (M/87)	ST147	*K. pneumoniae*	20/11/17	Y	U	Y	Y (this hospital, 1 month)	6 days	Not tested
Emergency	10 (F/94)	ST147	*K. pneumoniae*	07/12/17	N	U	N	Y (this hospital, >3 months)	13 days	+ (7 months)
Emergency	15 (F/79)	ST392	*K. pneumoniae*	18/12/17	Y	B	Y	Y (this hospital; 15 days)	6 days	+ (14 days)
Emergency	19 (F/79)	ST147	*K. pneumoniae*	29/12/17	Y	U	N	Y (this hospital; 1 month)	13 days	- (2 months)
Emergency	30 (M/74)	ST147	*K. pneumoniae*	12/02/18	Y	U	Y	N	10 days	Not tested
	30 (M/74)	ST147	*K. pneumoniae*	13/02/18						
Medical	11 (F/96)	ST307	*K. pneumoniae*	12/12/17	N	U	Y	Y (this hospital, 1 month)	16 days	+ (1 month)
Medical	18 (F/66)	ST15	*K. pneumoniae*	28/12/17	N	U	Y	N	45 days	+ (12 days)
Medical	20 (F/82)	ST22	*K. pneumoniae*	09/01/18	Y	U	Y	N	36 days	- (6 days)
Medical	22 (F/86)	ST147	*K. pneumoniae*	12/01/18	Y	U	Y	N	26 days	+ (1 day)
Medical	24 (M/85)	ST147	*K. pneumoniae*	22/01/18	Y	W	Y	N	28 days	+ (2 months)
Medical	31 (F/81)	ST392	*K. pneumoniae*	12/02/18	Y	U	Y	N	19 days	+ (2 days)
Medical	31 (F/75)	ST392	*K. pneumoniae*	21/02/18	N	U	Y	N	6 days	Not tested
Medical	6 (F/84)	ST392	*K. pneumoniae*	29/11/17	N	U	N	Y (this hospital, 1 month)	29 days	+ (1 month)
Medical	25 (M/42)	ST131	*E. coli*	25/01/18	N	W	Y	Y (another hospital)	50 days	+ (20 days)
Surgical	4 (F/60)	ST147	*K. pneumoniae*	21/11/17	N	W	Y	N	45 days	+ (7 days)
Surgical	5 (M/32)	ST147	*K. pneumoniae*	28/11/17	N	W	Y	Y (this hospital, 2 months)	22 days	+ (3 months)
Surgical	8 (F/91)	ST359	*K. pneumoniae*	04/12/17	N	R	Y	N	16 days	- (3 days)
Surgical	9 (F/48)	ST11	*K. pneumoniae*	05/12/17	N	U	Y	N	62 days	+ (5 days)
Surgical	12 (F/78)	ST147	*K. pneumoniae*	12/12/17	N	W	Y	Y (another hospital)	59 days	+ (1 month)
Surgical	13 (F/61)	ST147	*K. pneumoniae*	15/12/17	N	U	N	N	17 days	+ (1 day)
Surgical	14 (F/92)	ST147	*K. pneumoniae*	16/12/17	N	W	Y	N	36 days	+ (5 days)
Surgical	16 (M/69)	ST147	*K. pneumoniae*	19/12/17	N	B	Y	N	30 days	+ (1 day)
Surgical	17 (M/60)	ST147	*K. pneumoniae*	26/12/17	N	U	Y	N	25 days	+ (7 days)
Surgical	21 (F/82)	ST147	*K. pneumoniae*	11/01/18	N	U	Y	N	80 days	+ (1 month)
Surgical	23 (F/68)	ST147	*K. pneumoniae*	12/01/18	N	R	Y	N	40 days	+ (7 days)
Surgical	26 (M/73)	ND	*K. pneumoniae*	31/01/18	N	W	Y	N	30 days	+ (5 days)
Surgical	27 (F/82)	ST147	*K. pneumoniae*	03/02/18	N	W	Y	Y (this hospital, 2 months)	30 days	+ (1 month)
Surgical	28 (M/78)	-	*E. cloacae* complex	07/02/18	N	W	Y	Y (another hospital)	15 days	+ (1 day)
Surgical	29 (M/77)	ST147	*K. pneumoniae*	08/02/18	N	U	Y	N	39 days	+ (9 days)
Surgical	32 (M/78)	ST392	*K. pneumoniae*	12/02/18	Y	U	Y	N	57 days	+ (15 days)
Surgical	1 (M/79)	ST147	*K. pneumoniae*	04/11/17	N	U	Y	Y (another hospital)	41 days	+ (2 days)
UCI	7 (M/61)	ND	*K. pneumoniae*	03/12/17	Y	U	Y	Y (this hospital, 2 months)	8 days	+ (2 months)

^a^ Date of last colonization (when tested). ND = Not Done. N = No; Y = Yes; B = Blood; U = Urine; W = Wound; R = Respiratory.

**Table 3:** Performance of Fourier Transform infrared (FT-IR) typing and epidemiological data of carbapenemase-producing *K. pneumoniae* isolates from the studied hospital.

*n* (%)	*wzi* Alelle	K-Type by *wzi* Sequencing	K-Type by FT-IR ^a^	MLST	Source ^b^	Hospital Ward
54 (55)	*wzi*64	K14/K64	KL64	ST147	S (*n* = 34) C (*n =* 20)	MU (*n* = 25) SU (*n* = 23) ER (*n* = 6)
20 (20.4)	*wzi*187	KL27	FT1, 1 NT	ST392	S (*n* = 15) C (*n* = 5)	MU (*n* = 15) SU (*n*=3) ER (*n* = 2)
5 (5.1)	*wzi*9	K9	FT2	ST22	S (*n* = 4) C (*n* = 1)	MU (*n* = 4) SU (*n* = 1)
4 (4.1)	*wzi*173	KL102	FT3	ST307	S (*n* = 3) C (*n* = 1)	MU (*n* = 3) SU (*n* = 1)
3 (3.1)	*wzi*19	K19	1 K19, KL107 *, 1 NT	ST15	S (*n* = 3)	SU (*n* = 3)
2 (3.1)	*wzi*93	K60/KL112	KL112	ST15	S (*n* = 1) C (*n* = 1)	MU (*n* = 1) SU (*n* = 1)
2 (2)	*wzi*53	K53	FT4	ND	S (*n* = 1) C (*n* = 1)	SU (*n* = 2)
2 (2)	*wzi*50	KL15/KL17/KL51/KL52	FT5	ND	S (*n* = 1) C (*n* = 1)	MU (*n* = 1) ICU (*n* = 1)
2 (2)	*wzi*461	NA	NT	ND	S (*n* = 2)	SU (*n* = 2)
2 (2)	*wzi*236	KL10	NT	ST359	C (*n* = 1) S (*n*=1)	SU (*n* = 2)
1 (1)	*wzi*75	KL105	NT	ST11	C (*n* = 1)	SU (*n* = 1)
1 (1)	*wzi*102	K31	KL62 *	ND	S (*n* = 1)	SU (*n* = 1)

^a^ FT-IR types not recognized by the model were attributed arbitrary designations (FT1-FT5). ^b^ S = Rectal Screening; C = Clinical isolate; NT = Not Typeable; NA = Not attributable; ND = Not Done; * Incorrect prediction. MU = Medical Units; SU = Surgical Units; ER = Emergency Room; ICU = Intensive Care Unit.

**Table 4:** Characterization of CPE producers other than *K. pneumoniae.*

Species	*E. coli* Phylogenetic Group (Nº)	Carbapenemase (Nº)	Source (Nº)	Hospital Ward
*E. coli*	B2 (5) ^a^	KPC-3 (3) OXA-48 (1)	S (4) C (1)	*n* = 2 SU *n* = 3 MU
	A (2)	KPC-3 (2)	S (2)	*n* = 2 MU
	E (2)	KPC-3 (2)	S (2)	*n* = 1 MU *n* = 1 SU
	B1 (1)	KPC-3 (1)	S (1)	*n* = 1 MU
	F (1)	KPC-3 (1)	S (1)	*n* = 1 MU
*K. oxytoca*	NA (4)	KPC-3 (4)	S (4)	*n* = 3 SU *n* = 1 MU
*E. cloacae* complex	NA (6)	KPC-3 (5) VIM-1 (1)	S (4), C (1) S	*n* = 5 SU *n* = 1 MU
*C. freundii* complex	NA (1)	KPC-3 (1)	S	*n* = 1 SU

^a^ B2 *E. coli* producing KPC-3 belongs to ST131; S = Rectal Screening; C = Clinical Isolate; MU = Medical Units; SU = Surgical units; NA = Not applicable.
